# First-time orgasm in a young man with lifelong anorgasmia after flibanserin use: a case report

**DOI:** 10.1093/sexmed/qfad066

**Published:** 2024-01-11

**Authors:** Gal Saffati, Taher Naeem, Basil Kaaki, Mohit Khera

**Affiliations:** Scott Department of Urology, Baylor College of Medicine, Houston, TX 77030, United States; Scott Department of Urology, Baylor College of Medicine, Houston, TX 77030, United States; Scott Department of Urology, Baylor College of Medicine, Houston, TX 77030, United States; Scott Department of Urology, Baylor College of Medicine, Houston, TX 77030, United States

**Keywords:** anorgasmia, flibanserin, orgasmic dysfunction, male sexual dysfunction, case report

## Abstract

**Introduction:**

Anorgasmia is a poorly understood phenomenon defined as either a lifelong or acquired consistent inability to achieve ejaculation. Despite the prevalence of anorgasmia, there is currently no established treatment for the condition.

**Aims:**

To report a unique case of a patient with lifelong anorgasmia who was able to achieve his first orgasm with off-label use of flibanserin.

**Methods:**

The present case study relies on the patient’s self-report and a review of the relevant literature. The patient provided written informed consent.

**Results:**

A 28-year-old male presented to our office with complaints of lifelong anorgasmia, without any signs of erectile dysfunction. He reported good libido and energy levels and denied any urinary symptoms or history of depression. The patient failed medical management with numerous off-label medications, including bupropion and bremelanotide. Despite having received 4 or 5 sex therapy sessions over 3 months, the patient reported that this treatment approach was not effective. Off-label use of flibanserin was then initiated, and after 28 to 32 doses over 4 weeks, he achieved his first orgasm. Notably, the patient experienced nocturia and insomnia. The follow-up International Index of Erectile Function score marginally improved by 2 points without any improvement in the overall satisfaction subdomain.

**Conclusion:**

This case highlights the challenges of managing anorgasmia and anejaculation in a young male patient. A stepwise approach involving pharmacotherapy and sex therapy was not successful. However, the off-label use of flibanserin ultimately resulted in the patient achieving his first orgasm, albeit with some side effects. Further studies are needed to evaluate the efficacy and safety of flibanserin in men for this indication.

## Introduction

An orgasm is a fundamental component of human sexual function, and its absence or impairment can lead to distress and affect overall quality of life. Although the neurobiological processes underlying orgasm have been extensively studied, disorders of orgasm remain a challenging clinical problem. The orgasm is a complex neurobiological process that results from sexual activity and/or arousal. During ejaculation, the brain processes the sensation of pressure buildup within the posterior urethra, leading to seminal fluid emission and contraction of the periurethral musculature, which triggers an orgasm.^1^

Anorgasmia—a disorder characterized by a perceived absence of orgasm experience, regardless of whether physiologic concomitants of ejaculation occur—is a common form of orgasmic dysfunction.^2^ Primary anorgasmia, which persists throughout life, is a rare disorder arising from an individual’s first sexual experience.^1^ Despite being an uncommon complaint, primary orgasmic disorders in men have an estimated prevalence of 1.5 per 1000.^3^ However, owing to underreporting, the true prevalence is likely to be higher.

Flibanserin, a Food and Drug Administration (FDA)–approved medication, has been studied as a treatment for sexual dysfunction in pre- and postmenopausal women. It was first approved in 2015 with an indication to treat acquired, generalized hypoactive sexual desire disorder.^4^ Further postmarketing studies have suggested that this medication may also improve orgasmic function in women.^5^ However, research on the use of flibanserin in men is limited, and theoretically, it could be used as a treatment for the same condition. This case report describes a young male who experienced his first orgasm after flibanserin treatment and contributed to the limited data on the use of flibanserin in men. This case highlights the potential role of flibanserin in the treatment of male orgasmic dysfunction and underscores the need for further research in this area. This case report was deemed exempt, in accordance with institutional board review, although the patient provided full consent.

## Case presentation

**Figure 1:**
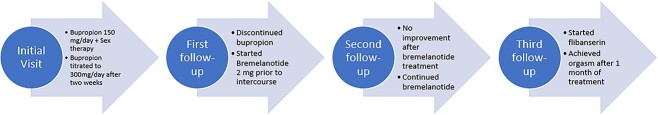
Summarized timeline of the patients’ clinical encounters.

A 28-year-old heterosexual male presented with lifelong anejaculation and anorgasmia without any signs of erectile dysfunction. The complete timeline of the patient’s clinical milestones is summarized in Figure 1. The patient denied any hematuria, dysuria, or urinary urgency or frequency and had a good urinary stream. There was no history of major surgery or any neurologic, infectious, or endocrine cause of anejaculation or anorgasmia. Additionally, the patient had no history of diabetes mellitus, multiple sclerosis, spinal cord injury, radical prostatectomy, hypogonadism, or hypothyroidism—all known causes of anorgasmia and anejaculation. The patient had no history of urethritis, genitourinary tuberculosis, or schistosomiasis. The patient was not taking any medications that could cause anorgasmia or anejaculation, such as alpha-methyldopa, thiazide diuretics, or antidepressants. Physical examination showed bilateral 16-mL descended testes, unilateral grade 2 palpable varicocele, and a normal circumcised phallus without any sensory dysfunction. The patient had prolactin and total testosterone levels of 12 ng/mL and 590 ng/dL, respectively. The baseline International Index of Erectile Function (IIEF) score was 41, with the following subdomain scores: erectile function, 23; intercourse satisfaction, 7; orgasmic function, 2; sexual desire, 5; and overall satisfaction, 4.

Initial treatment involved the prescription of bupropion at 150 mg/d, which was titrated to 300 mg/d after 2 weeks, with a complete psychological evaluation and sex therapy. However, the patient reported that bupropion and sex therapy were ineffective at the first follow-up. The medication was gradually weaned off by reducing the dosage to 150 mg for 1 week and then discontinuing it. The patient was then prescribed bremelanotide (10 mg/1 mL), with instructions to inject 0.2 mL (2 mg) at least 1 hour prior to intercourse. Ondansetron was administered as required for nausea. During his second follow-up, the patient indicated that he had used bremelanotide for the first time with no significant side effects. He was able to maintain an erection for more than an hour, but unfortunately, he was unable to achieve an orgasm despite trying several forms of stimulation. The patient experienced penile pain after this attempt. During the third follow-up, the patient reported using bremelanotide a total of 4 times, but it was ineffective, so the patient was prescribed flibanserin at 100 mg daily. After 28 to 32 doses of flibanserin, the patient stated that he had successfully reached orgasm and ejaculation during masturbation for the first time. Yet, he had trouble falling and staying asleep with this medication, so he was prescribed 50 mg of trazodone as needed. He also complained about drowsiness during the day as well as an increase in depressive symptoms. With continued use, the patient experience 2 more orgasms in a span of 1 month after the first orgasm, but he had an increase in side effects—namely, depressive and aggressive mood. The patient planned to continue trying the medication with a partner to evaluate its effectiveness. Follow-up IIEF scores were similar to baseline, with a total score of 43 and subdomain scores as follows: erectile function, 25; intercourse satisfaction, 7; orgasmic function, 2; sexual desire, 5; and overall satisfaction, 4.

## Discussion

This case report presents the unique situation of a 28-year-old male with lifelong anejaculation and anorgasmia. Despite thorough investigation, including medical history and physical examination, no apparent cause of anorgasmia was found.

The management of anorgasmia remains a therapeutic challenge given the paucity of FDA-approved medications and the less-than-optimal efficacy and unfavorable adverse event profiles of the agents studied. For example, in cases where medication-induced anorgasmia is suspected, cessation of implicated drugs, such as selective serotonin reuptake inhibitors, should be considered a first-line strategy. Currently, there is a need for FDA-approved pharmacotherapies for anejaculation, and the available literature on the existing pharmacotherapy is limited. Most published studies consist of case reports and nonrandomized, non–placebo-controlled case series. The 2020 American Urologic Association’s ejaculatory disorder guidelines proposed various off-label medications for addressing delayed ejaculation in men, including oxytocin, pseudoephedrine, ephedrine, midodrine, bethanechol, cabergoline, yohimbine, and imipramine. However, most of these drugs are infrequently used in urologic practice, and urologists should be mindful of the potential interactions and side effects associated with each medication. The patient’s assessment of the potential benefits of enhanced orgasmic function should be weighed against the possible risks.

In addition to the plethora of off-label pharmacologic therapies available to treat anorgasmia, sex therapy has been deemed an important treatment option for addressing this condition. For example, psychosexual counseling may be highly beneficial in cases of primary inhibited orgasm and may serve as adjunctive therapy in cases of delayed orgasm or ejaculation that are secondary to medical conditions. A comprehensive evaluation, encompassing the patient’s entire sexual history, was conducted. However, in our case, this information could not be obtained from the sexual therapy sessions, thereby limiting our ability to present the case in full. Moreover, while various psychotherapeutic treatments have been described for the management of delayed or anejaculation and some appear to be effective, none have undergone rigorous evaluation in large-scale samples.^6^ In the present case, sex therapy was ineffective.

To address our patient’s lifelong anejaculation and anorgasmia, the initial treatment involved the administration of bupropion and sex therapy. Notably, Abdel-Hamid and Saleh reported that use of bupropion (150 mg/d) over a 2-month period resulted in a 26% reduction in mean ejaculatory latency time and a corresponding increase in the percentage of patients who rated control over ejaculation as “fair to good” (from 0% to 21%) in a group of 19 age-matched males with lifelong delayed orgasm. Additionally, significant improvements were observed in the orgasm and intercourse satisfaction domain scores on the IIEF following treatment.^7^ Unfortunately, this approach failed to produce substantial improvements. Bremelanotide was then prescribed, which has been shown to be effective in treating anorgasmia in premenopausal women.^8^ Although bremelanotide helped the patient maintain an erection for an extended period, it did not result in orgasm.

Flibanserin, which is a serotonin 5-HT1A receptor agonist and 5-HT2A receptor antagonist, was prescribed for the patient.^9^ Although flibanserin is approved by the FDA for the treatment of hypoactive sexual desire disorder in premenopausal women, it is not approved for use in males.^4^ Nevertheless, the off-label use of flibanserin was considered in this case because of its potential to improve sexual function through its central effects on the serotonergic system and its proven improvement in orgasmic subdomain scores in the Female Sexual Function Index in women with hypoactive sexual desire disorder.^5^ After 28 to 32 doses of flibanserin, the patient achieved orgasm and ejaculation during masturbation for the first time. It has been shown that men and women share similar neural circuitry activation and deactivation during an orgasm. This process involves the activation of multiple brain zones, including the prefrontal cortex, as well as dopaminergic pathways from the ventral tegmentum to the nucleus accumbens.^10^ Owing to this shared circuitry, flibanserin may improve orgasmic function in men as well as women. Excitatory neurotransmitters such as dopamine and norepinephrine, which enhance orgasmic function, act in the prefrontal cortex. Conversely, inhibitory neurotransmitters that impair orgasmic function, such as serotonin, act in the same brain region. Evidence suggests that flibanserin increases the release of dopamine and norepinephrine, while reducing the release of serotonin in prefrontal brain areas. This is consistent with the same sites of abnormal neuroimaging findings described in patients with reduced sexual interest and desire. As such, the mechanism of action of flibanserin can be described as the downstream release of dopamine and norepinephrine and the reduction of serotonin in these brain circuits, thereby regulating reward processing in premenopausal women with reduced sexual interest and desire.^9^

The capacity for an orgasm involves organic and psychosocial factors, making it a complex process. Thus, the cumulative effect of successive treatments over an extended duration may have enabled the patient to address secondary influences that could have affected his ability to achieve orgasm.

## Conclusion

This case presents a challenging situation of lifelong anejaculation and anorgasmia in a young male with no apparent cause. The use of bupropion and sex therapy was not effective, and bremelanotide was ineffective in achieving an orgasm. The off-label use of flibanserin was eventually successful in achieving an orgasm and ejaculation in the patient, but further studies are required to validate its safety and efficacy in males. This case highlights the importance of considering less common causes of sexual dysfunction and exploring new treatment options when more common etiologies have been ruled out.

## Author contributions

Conception and design: G.S., T.N., B.K., M.K. Acquisition of data: G.S., T.N., M.K. Analysis and interpretation of data: G.S., T.N., M.K. Drafting the article: G.S. Revising it for intellectual content: G.S., T.N., B.K., M.K. Final approval of the completed article: G.S., T.N., B.K., M.K.

## Funding

None declared.

## Conflicts of interests

M.K.: consultant for Endo, AbbVie, Marius, Halozyme, Boston Scientific, Coloplast, Tolmar, and Petros; investor in Sprout. B.K.: consultant for Endo. The other authors declare no conflicts of interest.
